# Network of Interactions Between Gut Microbiome, Host Biomarkers, and Urine Metabolome in Carotid Atherosclerosis

**DOI:** 10.3389/fcimb.2021.708088

**Published:** 2021-10-07

**Authors:** Rui-Jun Li, Zhu-Ye Jie, Qiang Feng, Rui-Ling Fang, Fei Li, Yuan Gao, Hui-Hua Xia, Huan-Zi Zhong, Bin Tong, Lise Madsen, Jia-Hao Zhang, Chun-Lei Liu, Zhen-Guo Xu, Jian Wang, Huan-Ming Yang, Xun Xu, Yong Hou, Susanne Brix, Karsten Kristiansen, Xin-Lei Yu, Hui-Jue Jia, Kun-Lun He

**Affiliations:** ^1^Department of Geriatric Cardiology, the Second Medical Center & National Clinical Research Center for Geriatric Diseases, Chinese PLA General Hospital, Beijing, China; ^2^Beijing Key Laboratory of Chronic Heart Failure Precision Medicine, Chinese PLA General Hospital, Beijing, China; ^3^Department of Research, Beijing Genomics Institute (BGI)-Shenzhen, Shenzhen, China; ^4^China National GeneBank, Beijing Genomics Institute (BGI)-Shenzhen, Shenzhen, China; ^5^Shenzhen Key Laboratory of Human Commensal Microorganisms and Health Research, Beijing Genomics Institute (BGI)-Shenzhen, Shenzhen, China; ^6^Shenzhen Engineering Laboratory of Detection and Intervention of Human Intestinal Microbiome, Shenzhen, China; ^7^Department of Biology, Laboratory of Genomics and Molecular Biomedicine, University of Copenhagen, Copenhagen, Denmark; ^8^Department of Human Microbiome, School of Stomatology, Shandong University, Shandong Provincial Key Laboratory of Oral Tissue Regeneration, Jinan, China; ^9^Institute Marine Research (IMR), Bergen, Norway; ^10^James D. Watson Institute of Genome Sciences, Hangzhou, China; ^11^Department of Biotechnology and Biomedicine, Technical University of Denmark, Kongens Lyngby, Denmark; ^12^Macau University of Science and Technology, Macau, China; ^13^Analysis Center of Big Data, Medical Innovation Research Division, Chinese PLA General Hospital, Beijing, China

**Keywords:** gut microbiota, urine metabolomics, metabolic disease, integrative omics, carotid arteriosclerosis

## Abstract

Comprehensive analyses of multi-omics data may provide insights into interactions between different biological layers concerning distinct clinical features. We integrated data on the gut microbiota, blood parameters and urine metabolites of treatment-naive individuals presenting a wide range of metabolic disease phenotypes to delineate clinically meaningful associations. Trans-omics correlation networks revealed that candidate gut microbial biomarkers and urine metabolite feature were covaried with distinct clinical phenotypes. Integration of the gut microbiome, the urine metabolome and the phenome revealed that variations in one of these three systems correlated with changes in the other two. In a specific note about clinical parameters of liver function, we identified Eubacteriumeligens, Faecalibacteriumprausnitzii and Ruminococcuslactaris to be associated with a healthy liver function, whereas Clostridium bolteae, Tyzzerellanexills, Ruminococcusgnavus, Blautiahansenii, and Atopobiumparvulum were associated with blood biomarkers for liver diseases. Variations in these microbiota features paralleled changes in specific urine metabolites. Network modeling yielded two core clusters including one large gut microbe-urine metabolite close-knit cluster and one triangular cluster composed of a gut microbe-blood-urine network, demonstrating close inter-system crosstalk especially between the gut microbiome and the urine metabolome. Distinct clinical phenotypes are manifested in both the gut microbiome and the urine metabolome, and inter-domain connectivity takes the form of high-dimensional networks. Such networks may further our understanding of complex biological systems, and may provide a basis for identifying biomarkers for diseases. Deciphering the complexity of human physiology and disease requires a holistic and trans-omics approach integrating multi-layer data sets, including the gut microbiome and profiles of biological fluids. By studying the gut microbiome on carotid atherosclerosis, we identified microbial features associated with clinical parameters, and we observed that groups of urine metabolites correlated with groups of clinical parameters. Combining the three data sets, we revealed correlations of entities across the three systems, suggesting that physiological changes are reflected in each of the omics. Our findings provided insights into the interactive network between the gut microbiome, blood clinical parameters and the urine metabolome concerning physiological variations, and showed the promise of trans-omics study for biomarker discovery.

## Introduction

Systemic metabolism is regulated at numerous levels. Apart from direct interactions, the gut microbiota may indirectly interact with the host *via* metabolites, which may appear in circulation and the urine. Considering the complexity and the inter-dependency of biological systems, a holistic approach integrating multiple omics data may further the understanding of metabolic disease development.

The gut microbiota is a metabolic “organ” co-evolving with the host. Composed of hundreds of trillions of microbes (Qin et al., 2010), it is an ecological community where members compete, cooperate, and synergize with each other. This community not only passively adapts to the local biotic microenvironment but also actively interacts with the host. The gut microbiota affects the integrity of the gut barrier, extracts energy from food, provides bioactive compounds, regulates metabolic homeostasis and acts as a pro-inflammatory-anti-inflammatory rheostat (Tremaroli and Backhed, 2012). The composition and functional capacity of the gut microbiota may hence reflect the physiological state of the host, providing information of possible pathological conditions. This notion is supported by results from several case-control studies reporting on gut microbiota signatures that are linked to metabolic diseases, such as obesity and diabetes (Turnbaugh et al., 2006; Qin et al., 2012; Liu et al., 2017). Perturbations of the microbiota may cause alterations in the metabolite profile in circulation (Pedersen et al., 2016; Liu et al., 2017; Gu et al., 2017), and in addition, the compositional and functional features of the gut microbiota may also be detectable in the urine (Marcobal et al., 2013).

Extensive pieces of research have investigated the relationship between gut microbiota and a variety of immune systems and metabolic diseases, but, despite these advances, no direct evidence has established a direct and causal relationship between altered gut microbiota and these diseases. To address this question, we chose carotid atherosclerosis, which is systemic disease sharing metabolism, chronic inflammation, immune, infection, and senility, to combine data on the composition and functional potential of the gut microbiome with blood and urine metabolite profiles. These results may reveal novel associations with clinical phenotypes and thereby provide new knowledge of host-microbiome interactions in health and disease. Findings from the past decade have suggested that the structure and composition of the gut microbiota are associated with carotid atherosclerosis in humans and animal models (Jonsson and Backhed, 2017). Metabolites filtered or produced by gut microbiota, such as trimethylamine-N-oxide, short-chain fatty acids (SCFAs), and secondary bile acids, have been observed to affect the development of atherosclerosis (Wang et al., 2011; Wahlstrom et al., 2016; Chen et al., 2018). Although the commensal microbiota composition has emerged as a risk factor for cardiometabolic disease (Schroeder and Bäckhed, 2016), it remains controversial how the gut metagenome contributes to the development of atherosclerosis (Wright et al., 2000; Stepankova et al., 2010; Wang et al., 2011; Li et al., 2016) and in particular how the gut microbiota influences atherothrombotic processes.Taking advantage of a cohort of treatment-naïve subjects representing various clinical phenotypes, we performed an integrative trans-omics study combining blood biomarkers, fecal metagenomics and urine metabolomics. We found that microbiome was a poor biomarker for carotid atherosclerosis even in functional attributes. Trans omics analysis identified potential biomarkers characterizing different phenotypes, while integration of the three omics datasets revealed novel covariations and links. Community modeling further revealed the intimate interaction among the three systems, especially between the gut microbiota and the urine metabolome.

## Materials and Methods

### Study Cohort and Sample Collection

138 Chinese individuals (aged 32-76, including 76 males and 57 females) living in the north of China were recruited during physical examination in a carotid atherosclerosis screening program at the Chinese PLA General Hospital. None of them had been diagnosed with diseases before the inclusion, and subjects using antibiotics or receiving other medication within 1 month were excluded. Ultrasound examination of the carotid arteries was used to diagnose carotid arteriosclerosis. Blood tests on 124 clinical parameters were performed (**Additional File: Tables S1-3**). Fecal and urine samples were collected on the day of physical examination, immediately frozen and stored at -80°C until being further processed.

The study was approved by the Medical Ethical Review Committee of the Chinese PLA General Hospital and the Institutional Review Board at BGI-Shenzhen. Informed written consent was obtained from all participants.

### Metagenomic Sequencing, Binning, and Annotation

DNA was extracted from fecal samples using Qiagen QIAamp DNA Stool Mini Kit (Qiagen, Germany) according to the manufacturer’s instructions. Construction of a paired-end library with insert size of 350bp was performed according to the manufacturer’s instruction, and the DNA library was sequenced with PE reads of 2×100bp on the Illumina Hiseq2000 platform (Illumina, US). The sequencing reads were quality-controlled as described previously (Fang et al., 2018), and implementation of the pipeline is available at https://github.com/jiezhuye/cOMG. The raw sequences with low quality were filtered and trim by overall accuracy (OA) control strategy (Fang et al., 2018) using OAs1 (-Qsys = 33, -minLen= 30, -Scut=0.9, -Qcut=0.8). Then the high-quality reads were aligned to hg19 by SOAP2.22 (identity ≥ 0.9) to remove human-related reads by removeHost (-D 4 -s 30 -r 1 -v 7 -i 0.9). After quality control and host gene removal, clean reads were mapped to the 9,879,896 genes in the integrated gene catalog (IGC, Li et al., 2014) with a threshold of more than 90% identity over 95% of the length by SOAP2.22. Gene abundances were determined as previously described (Li et al., 2014), and subsequently used to calculate the relative abundances of genera and KEGG Orthology (KOs). The genes were clustered into Metagenomic species (MGS) based on co-abundance and gene content (≥700 genes) (Nielsen et al., 2014) with MGS canopy algorithm (Nielsen et al., 2014). Taxonomy assignment was based on >50% of the genes in one MGS (Qin et al., 2012). The relative abundance of a MGS was calculated from the mean relative abundance of its constituent genes (Qin et al., 2012). MGS present in >10% samples were subjected to further analysis (**Additional File: Table S4**).

### Correlation Analysis Between the Gut Microbiome, the Urine Metabolome and Blood Biomarkers

Spearman correlation was determined between the microbiome (relative abundance of MGS or functions) and blood biomarkers, between the urine metabolome (metabolite groups) and blood biomarkers (**Additional File: Table S5**), and between the urine metabolome and the gut microbiome using the cor function in the base R package. MGS/functions, metabolite groups, and biomarkers that reached statistical significance at three rounds of correlations were retained (p<0.05), and correlations with q<0.1 are indicated in the heatmap.

### Community Analysis

Pair-wise Spearman correlation was calculated between MGSs and urine metabolites, between urine metabolites and blood biomarkers, and between MGS and blood parameters (**Additional File: Table S6**) with base cor function in R software. Multiple hypothesis testing was adjusted using the method of Benjamini and Hochberg (q<0.1), and correlations were further filtered by a cutoff of |ρ|≥0.3. Only inter-omic correlations were used for community analysis. Non-directional communities composed of nodes from and edges between the three sets of omics data were modeled using the algorithm proposed by Girvan and Newman (Girvan and Newman, 2002) with igraph package in R. This method involved the iterative calculation of edge betweenness centrality on a network, which is the number of weighted shortest paths from all vertices to all other vertices that pass over a given edge. After each iteration, the edges with the highest betweenness centrality were removed, and the process was repeated until only individual nodes remained. Community structure was visualized with igraph package in R (**Additional File: Tables S7-8, S10**).

### Prediction and Classification by Random Forest

Prediction of clinical parameters was performed by a 5-repeated 10-fold down-sampling cross-validation random forest model (R 3.3.0, random Forest 4.6-12 package) using the relative abundance of MGS as input. Downsample majority class was controlled by the sampsize and strata parameter in the randomForest function. Prediction accuracy was evaluated by Spearman correlation between the predicted value and the measured value.

Non-carotid atherosclerosis versus carotid atherosclerosis classification was performed by a 5-repeated 10-fold down-sampling cross-validation random forest model (R 3.3.0, random Forest 4.6-12 package) using blood biomarkers or MGSs as input (**Additional File: Tables S9, S11**). Five-repeated 10-foldcross-validationis a robust model matric estimation and down-sampling to deal with the unbalanced sample size between groups. Receiver operator characteristic (ROC) curves were plotted using this classifier with R 3.3.0, pROC package.

### Functional Analysis of Microbiota

The reporter score (Z-score) (Patil and Nielsen, 2005) was calculated for KO modules or the gut metabolic modules (GMMs) (Vieira-Silva et al., 2016) to identify the differentially enriched functions comparing non-carotid atherosclerosis and carotid atherosclerosis groups. Modules with |Z-score|≥1.96 were considered significant.

### Urine Metabolomics

Metabolomic profiling of urine samples was performed on a 2777C UPLC system (Waters, UK) coupled to a Xevo G2-XS QTOF mass spectrometer (Waters, UK). Peak picking and compound identification were performed with Progenesis QI (ver2.2), aligned against the HMDB (The Human Metabolome Database) and KEGG (The Kyoto Encyclopedia of Genes and Genomes) databases. The untargeted metabolite data set was subjected to dimension reduction by the cluster of co-abundant metabolites into groups following the methods provided by a previous study using the R package Weighted gene co-expression network analysis (WGCNA) (Pedersen et al., 2016). The positively charged and negatively charged metabolites were analyzed together. Signed weighted co-abundance correlations (biweight midcorrelations after log2 transformation) were calculated across all individuals. A scale-free topology criterion was used to choose the soft threshold of β = 13. Clusters were identified with the dynamic hybrid tree-cutting algorithm, using deep split of 4 and a minimum cluster size of 3. The profile of each metabolite cluster was summarized by the cluster eigenvector.

## Results

### Characteristics of the Study Cohort and Clinical Parameters

We included 138 treatment-naïve subjects who presented wide variations in clinical blood parameters (based on 124 blood biomarkers, including biomarkers associated with metabolic abnormalities such as hyperglycemia, hyperinsulinemia, hyperlipidemia, and kidney dysfunction) (**Additional File: Tables S2-3**). Amongst these treatment-naïve subjects, we have an over representation of individuals diagnosed with carotid atherosclerosis at the time of sampling (n=102), as the study subjects took part in a specifically designed screening program for carotid atherosclerosis. Due to their display of a diverse range of metabolic abnormalities and a large variation in blood biomarkers, the collected data allowed us to evaluate possible associations between gut microbiota composition, clinical parameters and urine metabolites across different clinical states.

### Selective Clinical Parameters Associated With the Gut Microbiota Profile

Shotgun-based metagenomic sequencing of fecal microbial DNA generated a total of 1110.22 Gb of data (on average 7.93 Gb data per sample), which we clustered into 645 MGS (Nielsen et al., 2014) present in more than 10% of samples.

**Figure 1:**
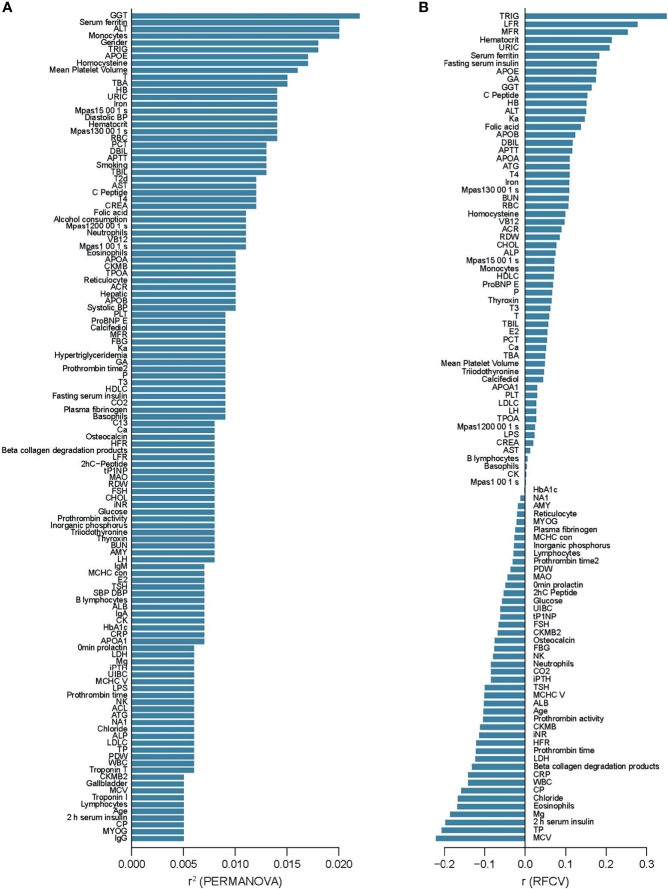
Alterations of the gut microbiome community associated with clinical parameters. **(A)** Clinical parameters that showed associations with MGS composition in PERMANOVA, ranked by the effect size (r^2^). p < 0.05, q < 0.1 **(B)** Prediction of clinical parameters by MGS relative abundances with random forest models, ranked by the performance (the correlation coefficient between predicted value and the measured value). p < 0.05.

To examine associations between phenotypes and the overall microbiota profile, we performed a permutational multivariate analysis of variance (PERMANOVA) of each variable vs MGS compositions. Thirty two variables were identified as covariates, of which gamma-glutamyl transferase (GGT, r^2^ = 0.022), ferritin (r^2^ = 0.020), alanine aminotransferase (ALT, r^2^ = 0.020), monocytes (r^2^ = 0.020), and triglyceride (TRIG, r^2^ = 0.018) had the largest effect sizes (p<0.05, q<0.1, **Figure 1A**). Five clinical covariates correlated significantly with α-diversity, and 6 clinical covariates correlated significantly with total gene counts in the gut microbiota (**Additional File: Figure S1**). Conversely, we also tested whether the microbiome data could be used to predict phenotypic variations. We built prediction models using random forest based on the relative abundance of MGSs. The gut microbiota-based predictor performed best in the prediction of triglyceride (TRIG) (r=0.35), followed by low fluorescence reticulocytes (LFR, r=0.28), middle fluorescent reticulocytes (MFR, r=0.25), hematocrit (r=0.21), and uric acid (URIC, r=0.21) (**Figure 1B**, and **Additional File: Figure S2**), indicating generally weak associations between certain blood biomarkers and the overall composition of the gut microbiota.

### Microbial Taxa Correlated With Clinical Phenotypes

**Figure 2:**
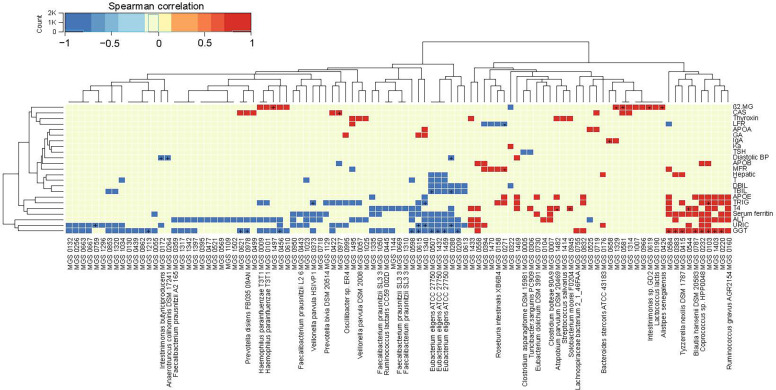
Correlations between the gut microbiome and clinical parameters. Heatmap showing correlations between MGS and clinical parameters (Spearman correlation, p < 0.05). ^+^q < 0.1; *q < 0.01. Color scale indicates the value of correlation coefficient.

We next aimed to identify potential biomarkers in the gut microbiome that could be linked to clinical parameters. Spearman correlation analysis was conducted between individual MGSs and clinical parameters. Among the false discovery rates (FDR)-adjusted significant correlations, the abundance of *Eubacteriumeligens* correlated negatively with GGT (MGS 0507, r=-0.30; MGS1459, r=-0.31; MGS1432, r=-0.33) and total blood levels bilirubin (TBIL) (MGS1459, r=-0.26; MGS1432, r=-0.28; MGS 0507, r=-0.33);, whereas the abundances of *Tyzzerellanexilis* (MGS0415, r=0.30) and *Blautiahansenii* (MGS0787, r=0.30) correlated positively with GGT. *Atopobiumparvulum* (MGS1482, r=0.39) and *Solobacteriummoorei* (MGS0945, r=0.35) correlated positively with blood levels of thyroxine (T4) (p<0.05, q<0.1, **Figure 2**). In line with this, *Eubacteriumeligens* and *Atopobiumparvulum* have been reported to be depleted and enriched, respectively, in patients with atherosclerotic cardiovascular disease (Jie et al., 2017). Accumulating evidence has shown that GGT, TBIL, and T4 serve as risk factors in carotid atherosclerosis by influencing host inflammation and oxidation stress, but relatively little is known regarding the mechanisms underlying the pathogenesis (Kozakova et al., 2012; Lee et al., 2020; Papadopoulou et al., 2020). We found a significant relation between GGT, TBIL, T4 and gut microbiota, which were involved in the progression of carotid atherosclerosis. Therefore, through an integrated analysis of multi-omics, we can further explore how gut microbiota contribute to the well-established link between these clinical parameters and carotid atherosclerosis.

### Microbial Functions Correlated With Clinical Phenotypes

Next, we analyzed the correlation between microbial functions and clinical parameters. The abundances of genes encoding proteins involved in amino acid metabolism (Proline biosynthesis, M00015, r=-0.27; Cysteine biosynthesis, M00021, r=-0.32; Tryptophan biosynthesis, M00023, r=-0.26; Histidine biosynthesis, M00026, r=-0.30; Ornithine biosynthesis, M00028, r=-0.31; Methionine biosynthesis, M00017, r=-0.28), gene expression (Aminoacyl−tRNA biosynthesis, M00359, r=-0.29; Aminoacyl−tRNA biosynthesis, M00360, r=-0.27; RNA polymerase, M00183, r=-0.30; Ribosome, M00178, r=-0.29; Ribosome, M00179, r=-0.30), and energy production (Coenzyme A biosynthesis, M00120, r=-0.27; NAD biosynthesis, M00115, r=-0.32; Pantothenate biosynthesis, M00119, r=-0.27) correlated negatively with monocyte counts, and the abundances of genes encoding proteins involved in tyrosine degradation (M00044, r=-0.31), and teichoic acid transport system (M00251,r=-0.30) correlated negatively with basophils. The abundances of genes involved in the transport of nickel and/or cobalt (M00246, r=0.37; M00245, r=0.36) positively correlated with iron (**Additional File 8: Figure S3**).

These correlations indicated that alterations in the microbiota taxa and function may be linked to specific physiological perturbations.

### Urine Metabolomic Variations Associated With Clinical Phenotypes

**Figure 3:**
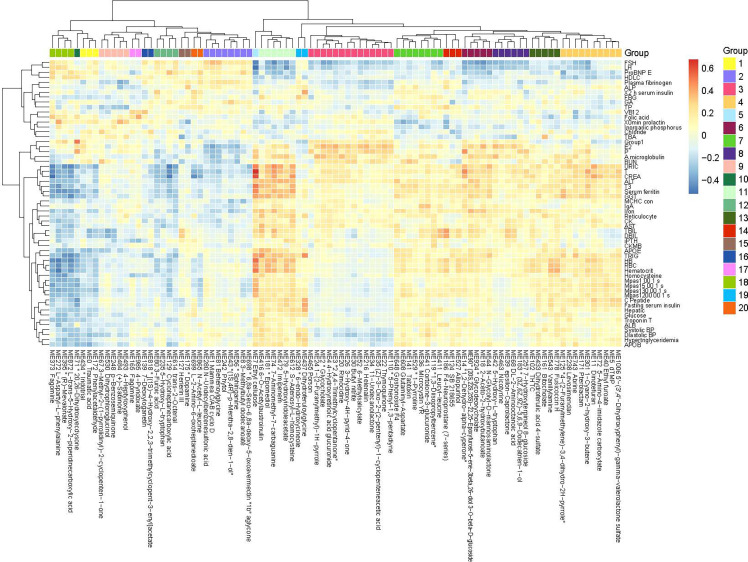
Correlations between the urine metabolome and clinical parameters. Heatmap showing correlations between urine metabolomic groups and clinical parameters (Spearman correlation, p < 0.05, q < 0.05). Only metabolites correlated significantly with more than one clinical parameter are included in the heatmap (the complete heatmap showing all correlations is illustrated in **Additional File 10: Figure S4**). Color scale indicates the value of correlation coefficient.

The metabolites in the urine may be generated by the host and/or the gut microbiota. By metabolome profiling of the urine, we detected 10890 positively charged and 9416 negatively charged metabolites, which were clustered into 1042 modules (more than 3 metabolites in each module) based on their co-abundance using the R package WGCNA (Pedersen et al., 2016). To assess their clinical relevance, we performed a correlation analysis between urine metabolites and clinical parameters. A large number of urine metabolites correlated with blood biomarkers, compartmentalized into distinct clusters exhibiting positive or negative correlations (**Figure 3** and **Additional File: Figure S4**). These urine metabolites demonstrated opposing correlation relationships with two sets of blood biomarkers. The metabolites that correlated positively with one set of blood biomarkers including follicle-stimulating hormone (FSH), luteotropic hormone (LH), glycated albumin (GA), brain natriuretic peptide brain natriuretic precursor (proBNP), high-density lipoprotein cholesterol (HDLC), vitamin B12 (VB12), calcifediol, beta-collagen degradation products, osteocalcin, total propeptide of type I procollagen (tP1NP), fibrinogen, and alkaline phosphatase (ALP), tended to correlate negatively with blood parameters related to liver function (URIC, creatinine [CREA], ALT, GGT, AST, TBIL, and direct bilirubin [DBIL]), blood pressure, lipid metabolism (apolipoprotein B [APOB], apolipoprotein E [APOE], TRIG), and glucose metabolism (C peptide, insulin, and glucose), and vice versa (**Figure 3** and **Additional File: Figure S4**). Among the phenotype-associated metabolites, those that correlated negatively with markers of liver dysfunction (e.g. URIC, CREA, ALT, GGT) included phenols, steroids, and possible food-derived compounds and metabolic intermediates (e.g. compounds structurally similar to fragomine, L−aspartyl−L−phenylalanine cycloheptanecarboxylic acid and, (R)−mevalonate, L−trans−5−hydroxy−2−piperidinecarboxylic acid, 20,26−dihydroxyecdysone), whereas metabolites that correlated positively included molecules that were possible metabolic intermediates or bioactive compounds (e.g. compounds structurally similar to ethyl cellulose, epomediol, 7−aminomethyl−7−carbaguanine, imipenem,S-adenosyl-L-homocysteine), suggesting the physiological involvement of those metabolites and related metabolic pathways. Thus, the urine metabolomic patterns reflected selective physiological features.

### Integration of the Microbiome Species, the Urine Metabolome and the Phenome

**Figure 4:**
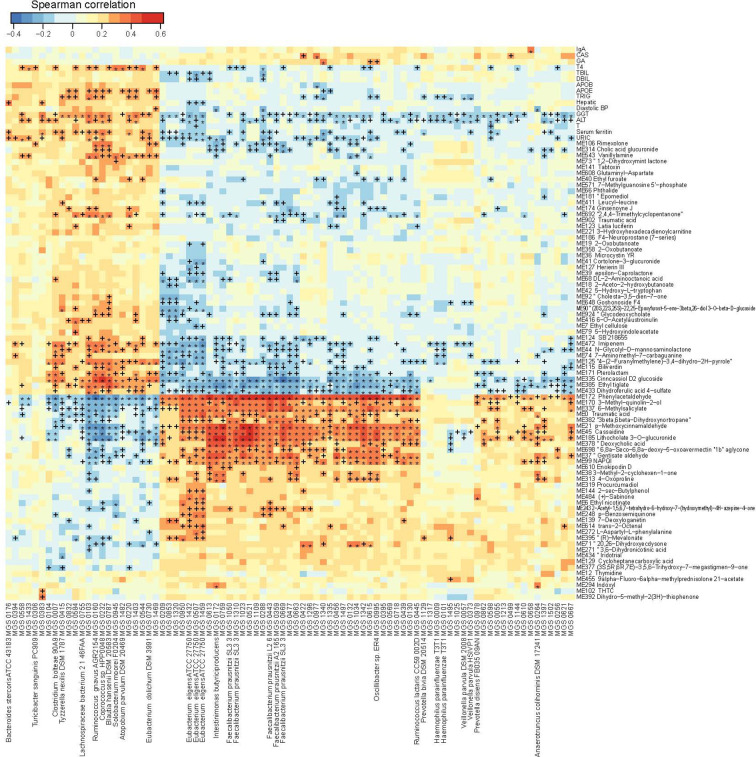
Trans-omics correlations between the gut microbiome, the urine metabolome and clinical parameters. Correlations that reached statistical significance (Spearman correlation, p < 0.05) between MGS and clinical parameters, between metabolites and clinical parameters, and between MGS and metabolites, are shown in the heatmap. ^+^q < 0.1; *q < 0.01. Color scale indicates the value of correlation coefficient.

To integrate data on the gut microbiome, the urine metabolome and the phenome, we performed three rounds of pairwise Spearman correlations, retaining triangular correlations for p<0.05 (**Figure 4**). Compartmentalized by their direction of correlation with clinical parameters, we found microbe-metabolite pairs showing positive correlations (which we termed disease-related microbes/metabolites) and negative correlations (which we termed health-related microbes/metabolites) with blood biomarkers where elevated levels have been associated with diseases (**Figure 4**). The correlation analyses indicated that for liver dysfunction-associated parameters (e.g. GGT and ALT), the disease-related bacteria included *Clostridium bolteae* (MGS0007), *Tyzzerellanexills*(MGS0415), *Ruminococcusgnavus*(MGS0160), *Blautiahansenii*(MGS0787), and *Atopobiumparvulum*(MGS1482), whereas the health-related bacteria included *Eubacteriumeligens*(MGS1432, MGS0507, and MGS1459), *Faecalibacteriumprausnitzii*(MGS1310), and *Ruminococcuslactaris*(MGS0445). These bacteria shared a collection of correlated health-related or disease-related metabolites; the disease-related metabolites included molecules structurally similar tocinncassiol D2 glucoside, ethyl tiglate, and dihydroferulic acid 4-sulfate, and the health-related ones included those similar to phenylacetaldehyde, 3-methyl-quinolin-2-ol, 6-methylsalicylate, p-methoxycinnarnaldehyde, cassaidine, lithocholate 3-o-gluronide, and gentisate aldehyde (**Figure 4**). Blood levels of APOE and/or TRIG correlated positively with the abundance of the disease-related bacteria *Ruminococcusgnavus*, *Blautiahansenii*, and *Atopobiumparvulum*,which in turn correlated with a series of urine metabolites. These included the health-related metabolites structurally similar to phenylacetaldehyde, 3-methyl-quinolin-2-ol and lithocholate, and the disease-related metabolites similar to cinncassiol D2 glucoside and ethyl tiglate.

### Integration of the Microbiome Function, the Urine Metabolome and the Phenome

Next, we applied the same inter-correlation approach to microbial modules, urine metabolites and blood biomarkers (p<0.05, q<0. 1). A few modules significantly correlated with URIC (e.g. modules in carbohydrate transport and metabolism, M00171, M00169, M00172, M00491, M00194, M00200, M00206, and M00201), and these modules correlated with the health-related metabolites (e.g. metabolites structurally similar to lithocholate 3-o-gluronide, methoxycinnarnaldehyde, or phenylacetaldehyde) and the disease-related metabolites (e.g. metabolites structurally similar to ethyl tiglate, dihydroferulic acid or imipenem).

Taken together, the results suggested that certain microbiome features, urine metabolites, and clinical parameters co-vary across treatment-naïve subjects.

### Organization of Trans-Domain Correlations Into One Two-Domain Tight-Knit and One Three-Domain Inter-Connected Communities

**Figure 5:**
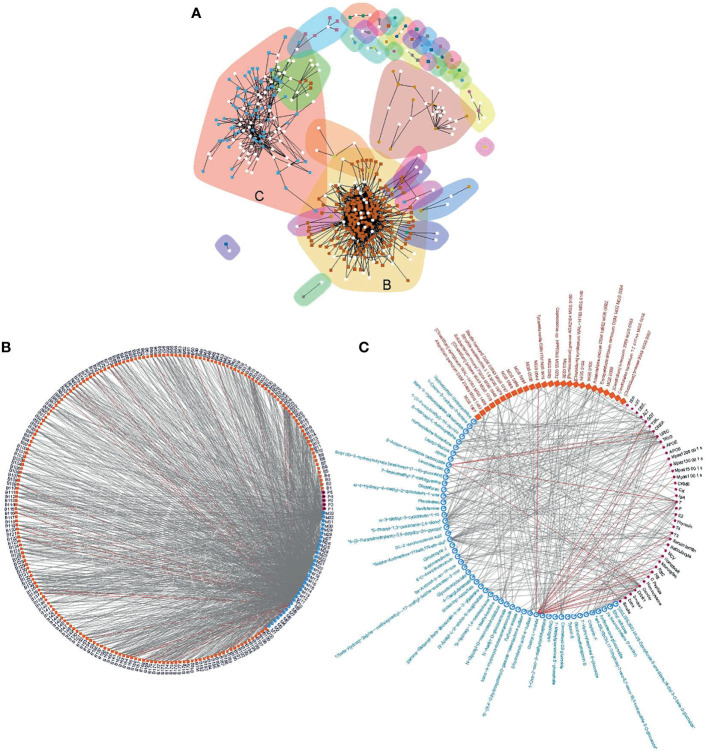
Correlation communities. Community modeling based on connectivity revealed two distinct communities **(A)**, including a large community composed of dense microbe-metabolite correlations **(B)**, and a microbe-metabolite-phenotype triangular correlated community **(C)**. Edge denotes correlation at q<0.1 and coefficient ≥ 0.3. The largest community **(B)** was formed by 1049 edges of which 99.1%were between MGS and metabolites (MGS and metabolites are coded, **Additional File 15: Table S10**). The second largest community **(C)** was formed by 270 edges where the majority of the three domains were connected. Grey lines, positive correlations; red lines, negative correlations.

Biological entities, such as microorganisms, exist in communities and interact with each other (Girvan et al., 2002). The interaction may be directly or indirectly *via* biomolecules. To unravel the structure of the interactive relationship between the gut microbiota, urine metabolites and clinical parameters, we modeled correlations with a previously reported algorithm based on edge betweenness (Girvan et al., 2002). After pruning the loose connections, two large communities emerged (**Figure 5A**). The largest community was densely weaved between 200 MGS and 33 metabolite groups (with 99.1%/1040 edges), partially linked to five blood biomarkers (with 0.2%/3 edges between MGS and phenotypes, and 0.7%/6 edges between metabolites and phenotypes, **Figure 5B**). This community represented a huge cluster of microbe-metabolite interactions, demonstrating the extensive linkage between the gut microbiota and the urine metabolome. The second-largest microbial community was formed by triangular correlations between 41 MGS, 53 metabolite groups and 23 blood biomarkers (with 5%/14 edges between MGS and phenotypes, 67%/181 edges between metabolites and phenotypes, and 28%/75 edges between MGS and metabolites), which was less dense than the first community (**Figure 5C**). In this community, entities from the three systems were all connected, where changes in one data set resulted in variations in the other two. The majority of inter-system correlations were positive, except that a metabolite structurally similar to diethyl L−malate correlated negatively with blood variables including GGT, URIC, TRIG, blood viscosity, testosterone (T), triiodothyronine (T3), ferritin, hematocrit, monocytes, red blood cell count (RBC), hemoglobin (HB), gender, alcohol consumption and smoking, and with one MGS (MGS0544), and that a metabolite structurally similar to 5−amino−4−imidazole carboxylate correlated negatively with CREA, URIC and T, indicating antagonistic or inhibitive relationships. These modeled communities demonstrated close host-microbiome interactions and trans-omics connections, especially between the gut microbiota and the urine metabolome.

### Towards the Identification of Carotid Atherosclerosis Biomarkers

**Figure 6:**
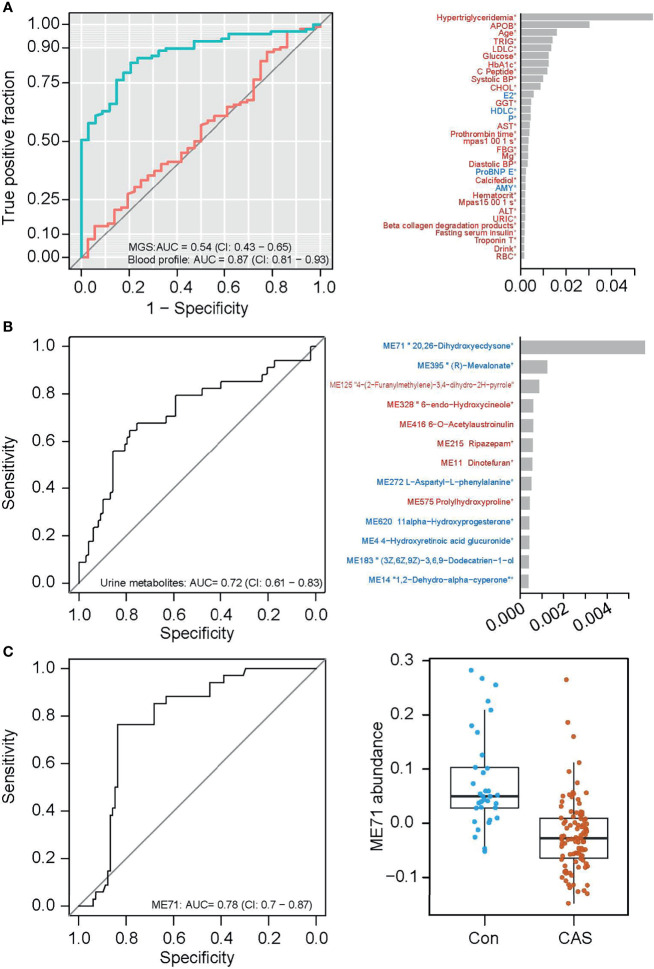
Classification of carotid atherosclerosis based on the gut microbiome or on the urine metabolome. **(A)** ROC curves of carotid atherosclerosis classification based on MGS relative abundances (red) and on the blood profile (green, the left panel), and the top important clinical parameters contributory to the prediction based on blood profile (the right panel). **(B)** The ROC curve of carotid atherosclerosis classification based on the urine metabolome (left panel), and the top important metabolite groups in the classification model (right panel). **(C)** The ROC curve of carotid atherosclerosis classification based on one urine metabolite ME71 (the left panel), and abundances of ME71 in the non-carotid atherosclerosis and carotid atherosclerosis groups (the right panel).

Carotid atherosclerosis is usually asymptomatic with high health risks, and an easily accessible biomarker for this disease is lacking (Tolle et al., 2015). In our cohort, the carotid atherosclerosis cases were identified based on ultrasound examination of the carotid arteries. We first compared the clinical parameters in groups with or without carotid atherosclerosis. Several variables differed significantly between individuals with and without carotid atherosclerosis. Principal coordinate analysis (PCoA) of the gut microbiota composition failed to distinguish between the microbiota of individuals with and without carotid atherosclerosis (**Additional File: Figure S7**), and the same was seen for microbial gene counts and α-diversity (**Additional File: Figure S8A**). Mann–Whitney U test identified a few MGS including *Prevotellacopri* and *Prevotellabivia* that differed in abundance between individuals with and without carotid atherosclerosis (p<0.05), but none of them passed the false discovery rate of q<0.1 (**Additional File: Figure S8B**). After gene mapping to both KEGG modules and GMM (Vieira-Silva et al., 2016), we found several functions altered in the relative gene abundance in the microbiota of carotid atherosclerosis individuals (**Additional File: Figure S9**). These included the reduction in the abundance of genes involved in methanogenesis and the enrichment of genes involved in the metabolism of low molecular mass carbohydrates in carotid atherosclerosis (**Additional File: Figure S9**). We also attempted to pinpoint the carotid atherosclerosis-related microbial features using a classification model. However, the 5-repeat 10-fold cross-validation random forest model performed better on blood biomarkers (AUC=0.87) than on MGS composition (AUC=0.54, **Figure 6A**), indicating a more marked deviation associated with carotid atherosclerosis in the blood profile than in the microbiota. However, since the blood-based discriminators (such as hypertriglyceridemia, APOB, TRIG, LDLC, and HbA1c) are common denominators for metabolic diseases, these are poor biomarkers for carotid atherosclerosis on their own. These results suggested that the gut microbiota in carotid atherosclerosis subjects exhibited only minor alterations, and is not useful on its own as a disease biomarker.

Urine has also previously been used as an easily accessible biomarker for several diseases (Bouatra et al., 2013). In search of potential urine biomarkers for carotid atherosclerosis, we likewise conducted feature selection using the 5-repeat 10-fold cross-validation random forest. The model classified individuals with and without carotid atherosclerosis with an area under the curve (AUC) of 0.72 based on selected urine biomarkers (**Figure 6B**). Notably, the mere inclusion of the top important metabolite group ME71 (structurally similar to 20, 26-dihydroxyecdysone) in the classification model reached an AUC of 0.78 (**Figure 6C**, the left panel). This group of metabolites was significantly lower in individuals with vs without carotid atherosclerosis (**Figure 6C**, the right panel). However, the variation of ME71 within each group suggests the need for further validation of its use as a biomarker for carotid atherosclerosis.

## Discussion

In the context of the diverse clinical phenotypes in our cohort, we integrated multi-omics data by characterizing inter-domain correlation relationships and further modeled the correlations into communities. The results showed close interactions between the blood profile, the urine metabolome and the gut microbiota, especially that the urine metabolome and the gut microbiota appeared to covary.

One prerequisite of an integrative trans-omics study is that there must be variations in the data points. The metabolic variations between subjects in this study ensured the variation of clinical variables and thereby the related omics, hence providing the necessary preconditions for an integrative study. In addition, the treatment-naïve nature of this cohort eliminated confounding by medication.

Our study, like many previous studies, indicates that changes in the abundance of specific bacteria correlate with fluctuations in certain clinical parameters. These bacteria may serve as putative disease biomarkers and provide potential therapeutic targets. Consistent with findings in our study, the genus *Eubacterium* is enriched in healthy controls as compared to patients with liver cirrhosis (Qin et al., 2014), and the abundance of *Ruminococcus* has been associated with liver fibrosis (Boursier et al., 2016). In mouse models, the administration of *Faecalibacteriumprausnitzii* has been shown to improve liver health and reduce hepatic fat accumulation (Munukka et al., 2017). In comparison to the gut microbiota, our results suggest that alterations of the urine metabolome may greatly co-vary with clinical parameters, and certain groups of urine metabolites may be generally linked to health or disease. These co-varying urine metabolites may be produced or regulated by the same or related metabolic pathways or by specific bacteria.

By conduction a trans-omic network analysis of all three data sets, we provide a view into the pattern of connections between the blood parameters, the gut microbiome, and the urine metabolome. The statistical correlation between the clinical parameters, the gut microbiome, and the urine metabolome does not permit distinction between direct biological crosstalk or co-occurrence. Nonetheless, association relationships may allow for the generation of hypotheses to be tested in further studies focusing on mechanisms or etiology. For instance, the metabolic characteristics of certain bacteria (for example, the health-related bacterium *Eubacteriumeligens* and the disease-related *Ruminococcusgnavus* are both degraders of complex carbohydrates (Mahowald et al., 2009; Crost et al., 2016) is one point worthy of further exploration for the potential mechanisms, and they may leave metabolite fingerprints in the blood and also in the urine that have functional impacts on the host (for example, the well-documented metabolic effects of short-chain fatty acids (Morrison and Preston, 2016). The two large communities uncovered by network modeling demonstrate that trans-system relationships are quite clustered and packed, suggesting that entities in the three systems interact frequently. The largest community highlighted the close association between the gut microbiome and the urine metabolome, and therefore the urine metabolomic profile at least in part may reflect the gut microbiota composition. The trans-omic network probably may be different between CAS and control. However, due to the small sample size of the control, we could not provide robust comparisons of trans-omics network between CAS and control here.

In a previous study, potential microbial biomarkers for atherosclerotic cardiovascular disease were identified based on 218 cases and 187 controls (Jie et al., 2017). However, our study indicates that carotid atherosclerosis is only associated with minor alterations of the gut microbiota. Further studies are needed to identify possible associations between the gut microbiota and carotid atherosclerosis, with adjustment of confounding factors and larger sample size. Notably, our findings suggest that the urine metabolome may contain promising biomarkers for carotid atherosclerosis, and the potential of the metabolite (ME71, structurally similar to 20, 26-dihydroxyecdysone, 2026E) as a carotid atherosclerosis marker warrants further validation. 2026E is less reported. But its end metabolites, 20-Hydroxyecdysone, have revealed many potential beneficial health effects including the wound-healing, immunoprotective and anti-osteoporosis effects in many studies (Buniam et al., 2020). 20-Hydroxyecdysone ameliorates metabolic and cardiovascular dysfunction in high-fat-high-fructose-fed ovariectomized rats (Buniam et al., 2020).

## Conclusions

Our results provide evidence that changes in clinical features related to the disease can be manifested as quantitative changes in multiple body systems including the blood profile, the gut microbiome, and the urine metabolome. These multiple systems are intertwined into closely linked networks. The precise definition and accurate classification of health and disease entail a data-driven, multi-omics and integrative approach for ultimate identification of the most consistent and reliable biomarkers for a given disease.

## Data Availability Statement

The datasets presented in this study can be found in online repositories. The names of the repository/repositories and accession number(s) can be found below: http://ftp.cngb.org/pub/CNSA/data1/CNP0000048/.

## Ethics Statement

The studies involving human participants were reviewed and approved by Medical Ethics Committee of Chinese PLA General Hospital. The patients/participants provided their written informed consent to participate in this study. Written informed consent was obtained from the individual(s) for the publication of any potentially identifiable images or data included in this article.

## Author Contributions

K-LH, QF, and R-JL designed the study. R-JL, C-LL, and Z-GX recruited subjects, collected samples, and contributed metadata. K-LH, LM, KK, YH, XX, H-MY, JW, and H-JJ supervised the project. R-JL, Z-YJ, X-LY, H-JJ, R-LF, FL, YG, H-HX, H-ZZ, BT, and J-HZ interpreted the data, prepared the display items and wrote the manuscript. All authors contributed to the article and approved the submitted version.

## Funding

This research was supported by the National Key Technologies R&D Program for New Drug of China (2018ZX09J18109-004), and by the Shenzhen Municipal Government of China (JSGG20160229172752028 and JCYJ20160229172757249).

## Conflict of Interest

The authors declare that the research was conducted in the absence of any commercial or financial relationships that could be construed as a potential conflict of interest.

## Publisher’s Note

All claims expressed in this article are solely those of the authors and do not necessarily represent those of their affiliated organizations, or those of the publisher, the editors and the reviewers. Any product that may be evaluated in this article, or claim that may be made by its manufacturer, is not guaranteed or endorsed by the publisher.

## Supplementary Material

Supplementary Figure 1 Correlation between Shannon diversity **(A)** or gene counts **(B)** with clinical variables.

Supplementary Figure 2 Prediction of clinical variables by MGS relative abundances with random forest models. Prediction accuracy was evaluated by the correlation between predicted value and the measured value.

Supplementary Figure 3 Correlation between microbial functional modules and clinical variables. Heatmap showing correlations between functional modules and clinical variables (spearman correlation, p < 0.05). ^+^q < 0.1; *q < 0.01. Color scale indicates the value of correlation coefficient.

Supplementary Figure 4 Correlation between urine metabolome and clinical variables. Heatmap showing correlations between functional modules and clinical variables (spearman correlation). Color scale indicates the value of correlation coefficient.

Supplementary Figure 5 Trans-omic correlation between microbial functional modules, the urine metabolome and clinical variables. Correlations between microbial functions and clinical variables, between urine metabolites and clinical variables, and between microbial functions and urine metabolites (spearman correlation, p < 0.05) are shown in the heatmap. ^+^q < 0.1; *q < 0.01. Color scale indicates the value of correlation coefficient.

Supplementary Figure 6 Clinical phenotypes were different between non-CAS and CAS groups. Wilcoxon rank-sum test was performed between non-CAS and CAS groups.

Supplementary Figure 7 PCoA of the genus composition in the gut microbiota. non-CAS, blue circles; CAS, red triangles.

Supplementary Figure 8 Mild alterations of the gut microbiota composition in CAS. **(A)** Comparison of gene counts (the left panel) and a-diversity (the right panel) of the gut microbiota in non-CAS and CAS individuals by Wilcoxon rank-sum test. **(B)** MGS differing in abundance between non-CAS and CAS at p<0.05 but q>0.1.

Supplementary Figure 9 Functional alterations of the gut microbiota in CAS. Differentially enriched modules at Z-score>1.6 as analyzed by KEGG **(A)** and by GMM **(B)** are shown. Green, enriched in the non-CAS group; red, enriched in the CAS group. Bar length indicates the value of reporter score.
