# Supplementary figures and images for: Network of Interactions Between Gut Microbiome, Host Biomarkers, and Urine Metabolome in Carotid Atherosclerosis
Source: Front Cell Infect Microbiol. 2021 Oct 7;11:708088. doi: 10.3389/fcimb.2021.708088 (PMC8529068; doi:10.3389/fcimb.2021.708088)

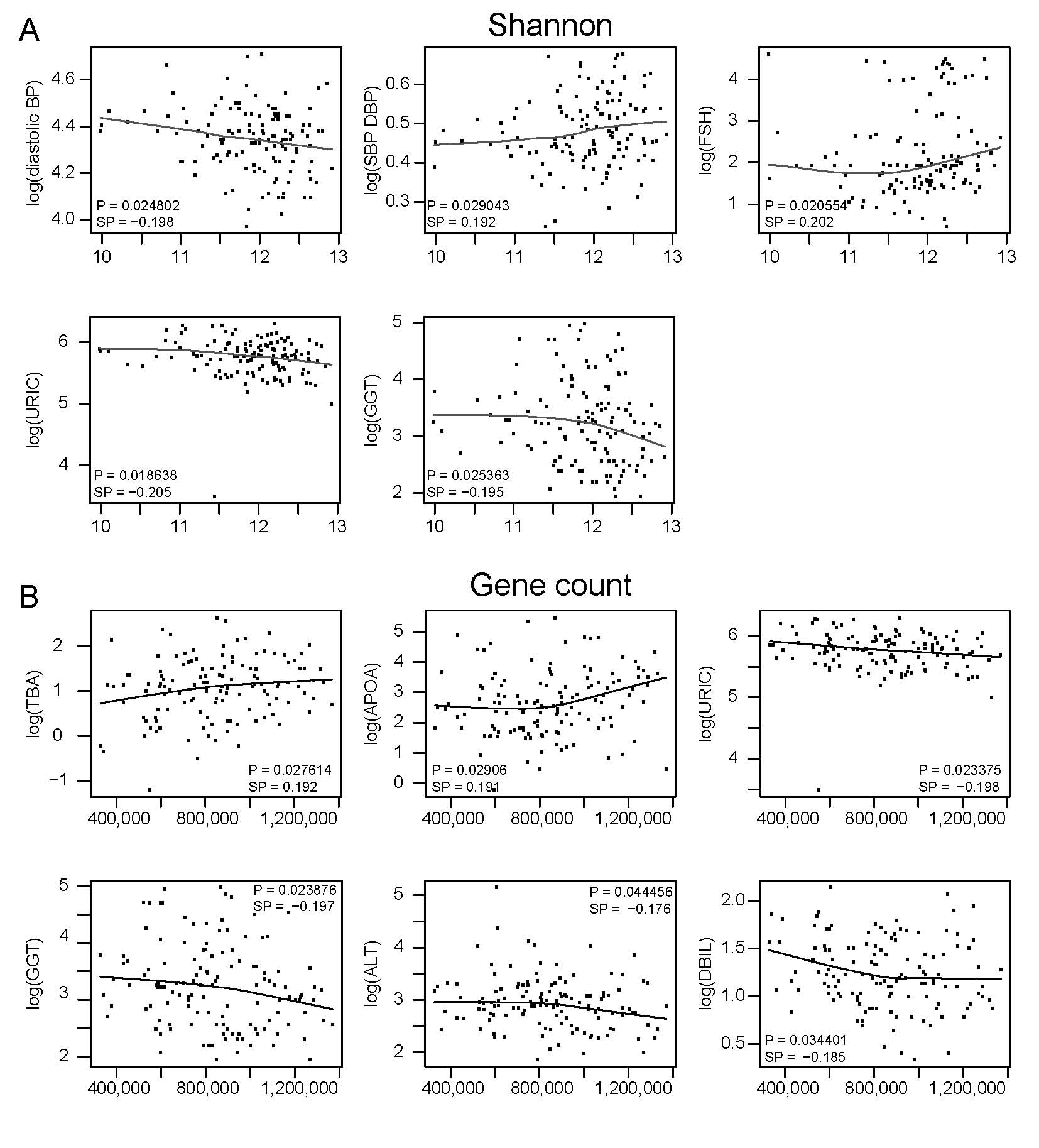

Supplement: Supplementary Figure 1 — Correlation between Shannon diversity (A) or gene counts (B) with clinical variables. [file Image_1.jpeg]

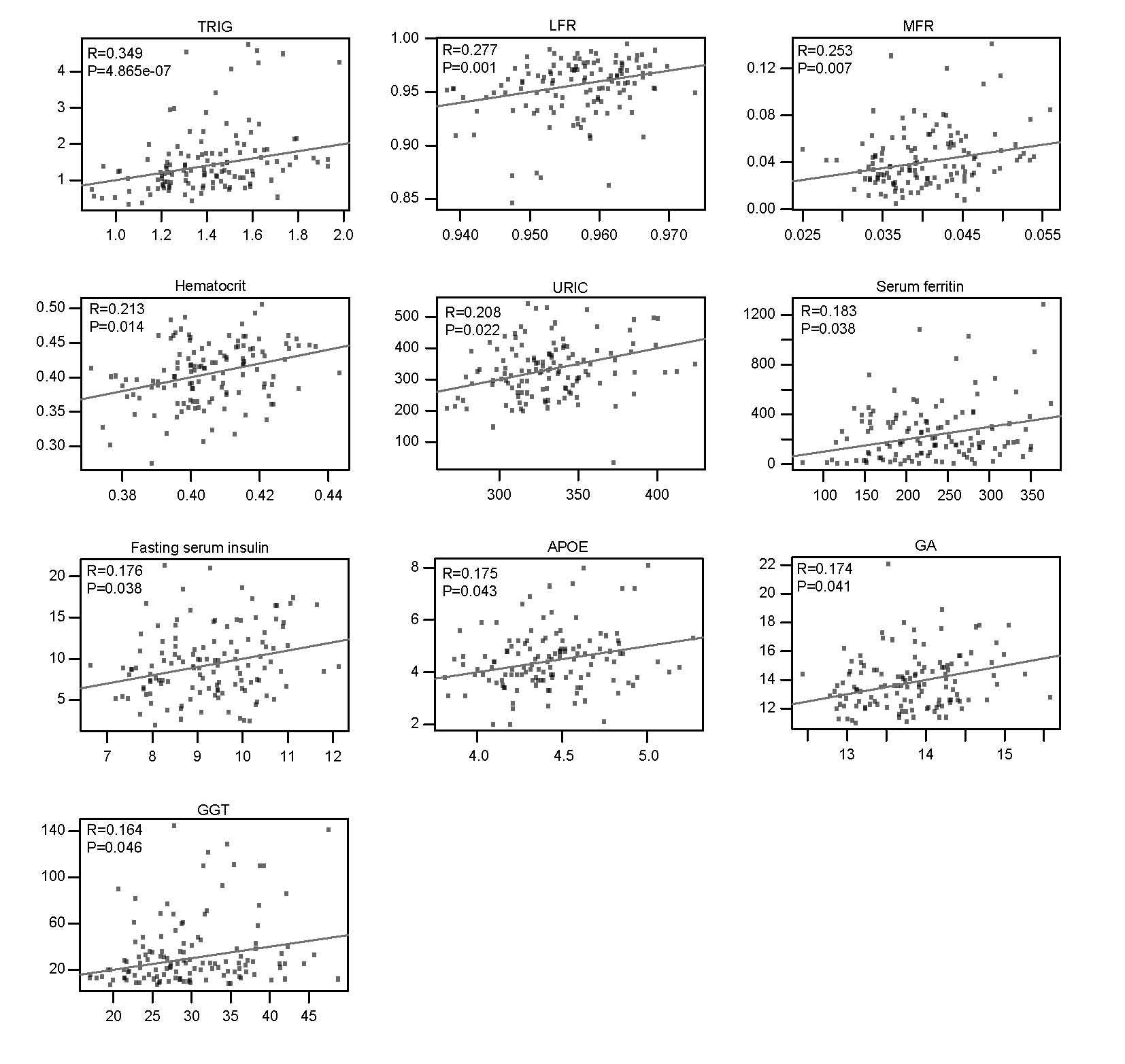

Supplement: Supplementary Figure 2 — Prediction of clinical variables by MGS relative abundances with random forest models. Prediction accuracy was evaluated by the correlation between predicted value and the measured value. [file Image_2.jpeg]

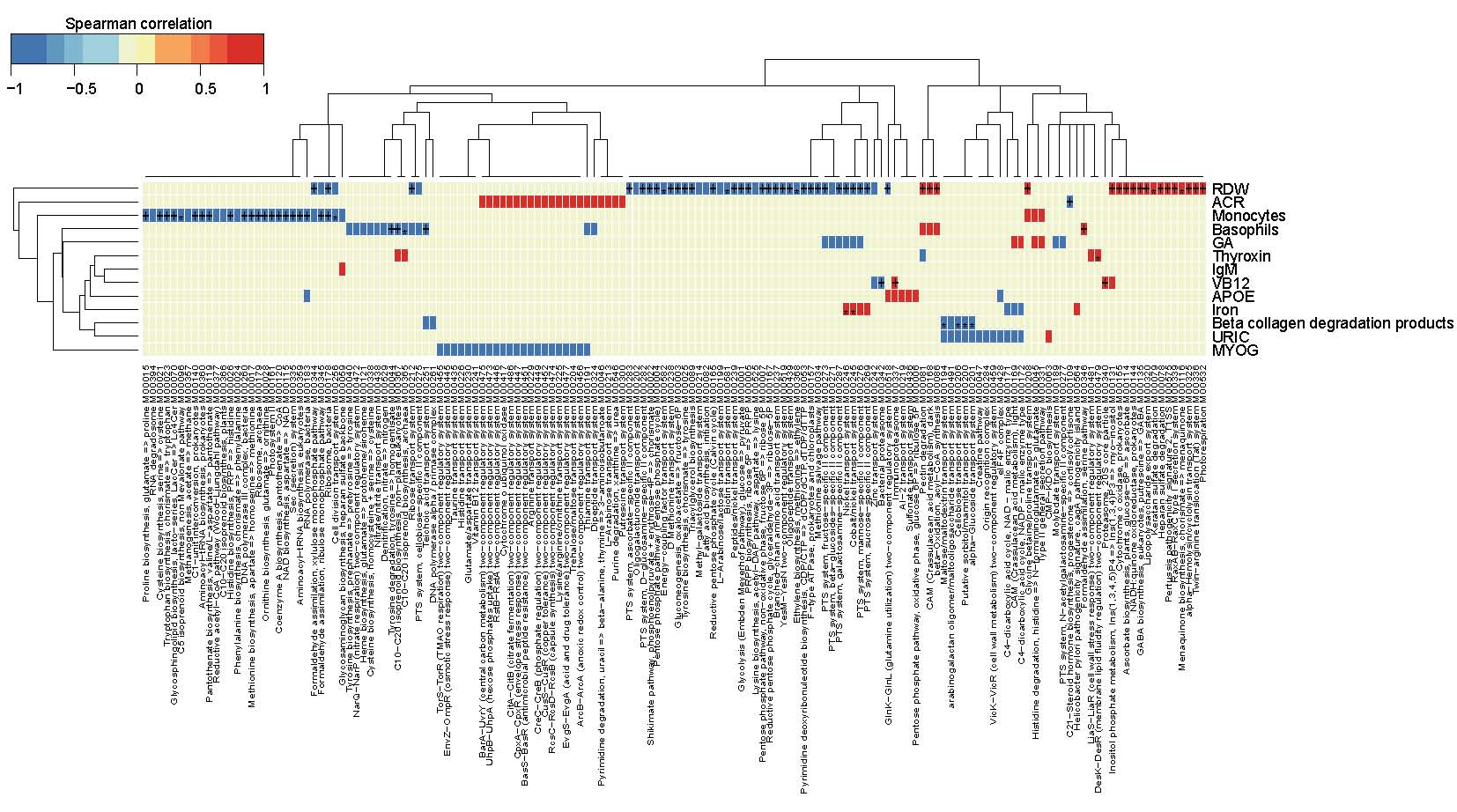

Supplement: Supplementary Figure 3 — Correlation between microbial functional modules and clinical variables. Heatmap showing correlations between functional modules and clinical variables (spearman correlation, p < 0.05). +q < 0.1; *q < 0.01. Color scale indicates the value of correlation coefficient. [file Image_3.jpeg]

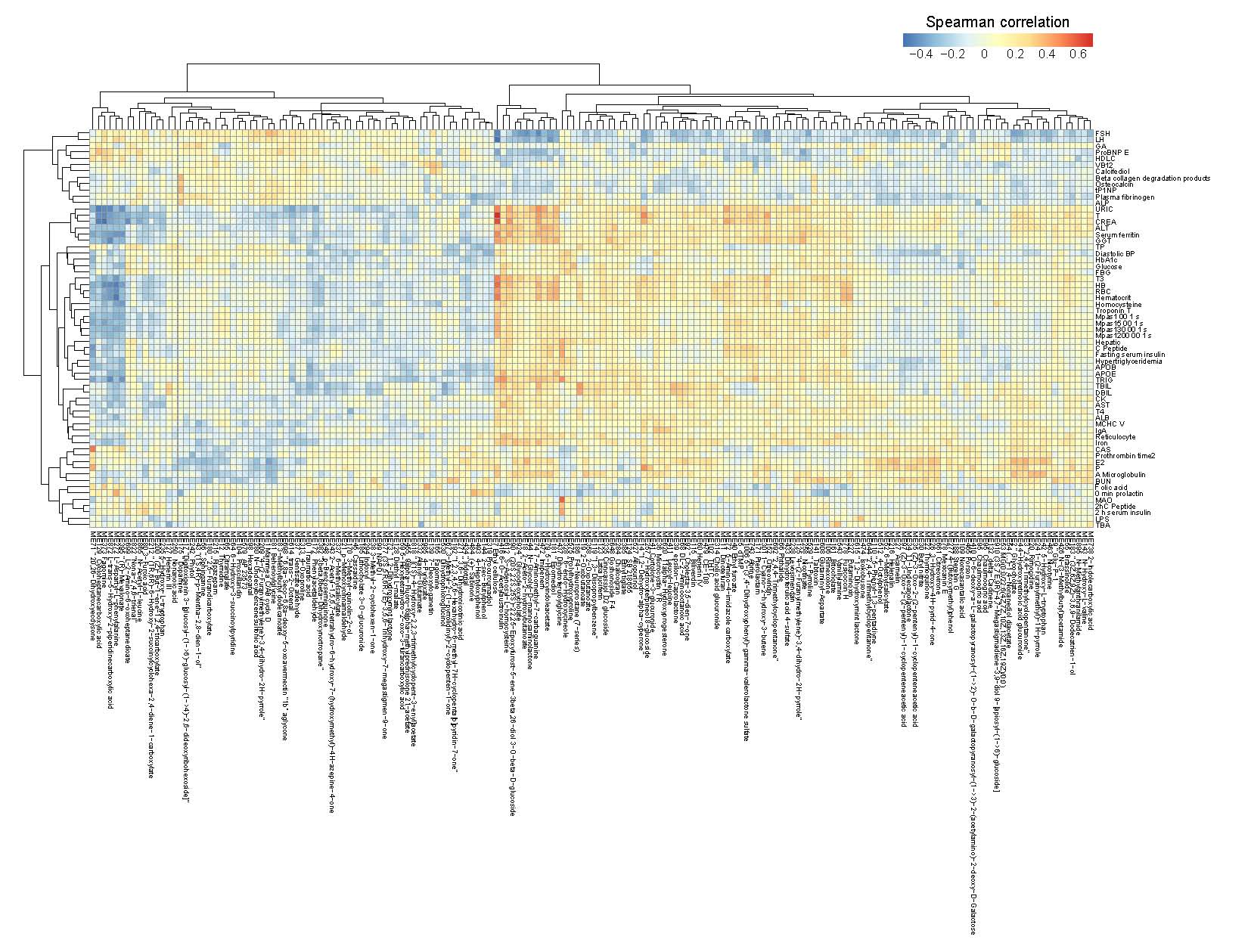

Supplement: Supplementary Figure 4 — Correlation between urine metabolome and clinical variables. Heatmap showing correlations between functional modules and clinical variables (spearman correlation). Color scale indicates the value of correlation coefficient. [file Image_4.jpeg]

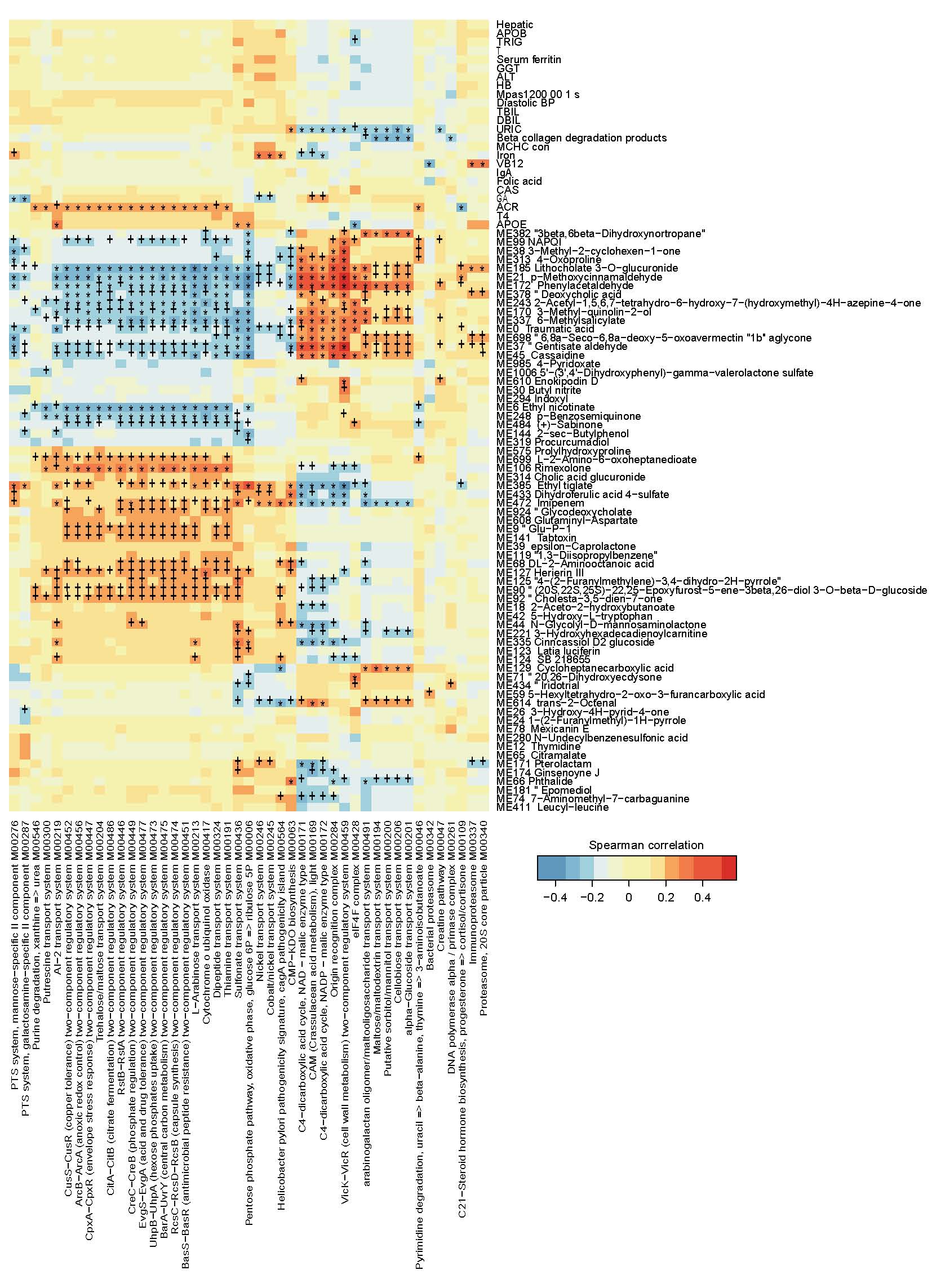

Supplement: Supplementary Figure 5 — Trans-omic correlation between microbial functional modules, the urine metabolome and clinical variables. Correlations between microbial functions and clinical variables, between urine metabolites and clinical variables, and between microbial functions and urine metabolites (spearman correlation, p < 0.05) are shown in the heatmap. +q < 0.1; *q < 0.01. Color scale indicates the value of correlation coefficient. [file Image_5.jpeg]

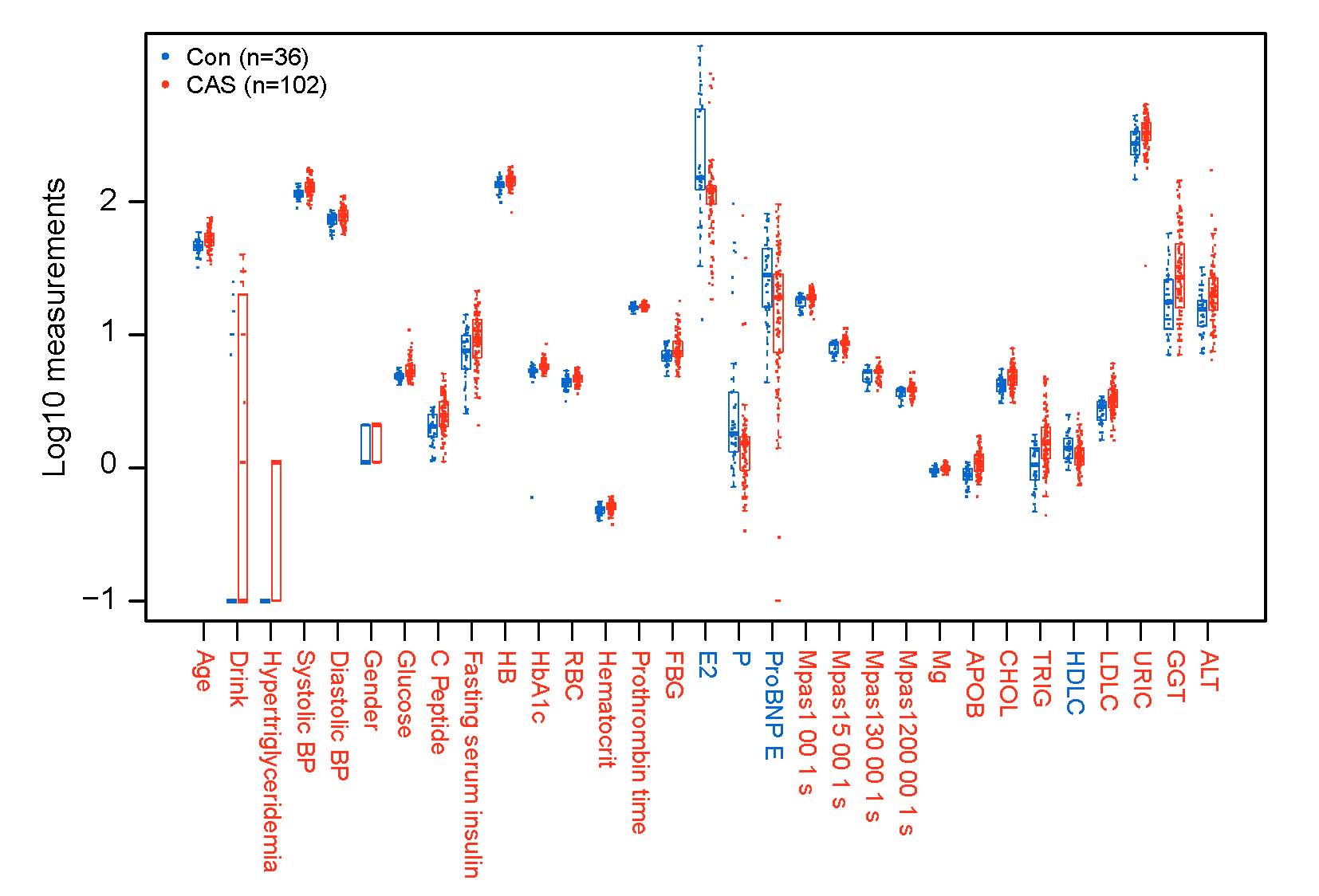

Supplement: Supplementary Figure 6 — Clinical phenotypes were different between non-CAS and CAS groups. Wilcoxon rank-sum test was performed between non-CAS and CAS groups. [file Image_6.jpeg]

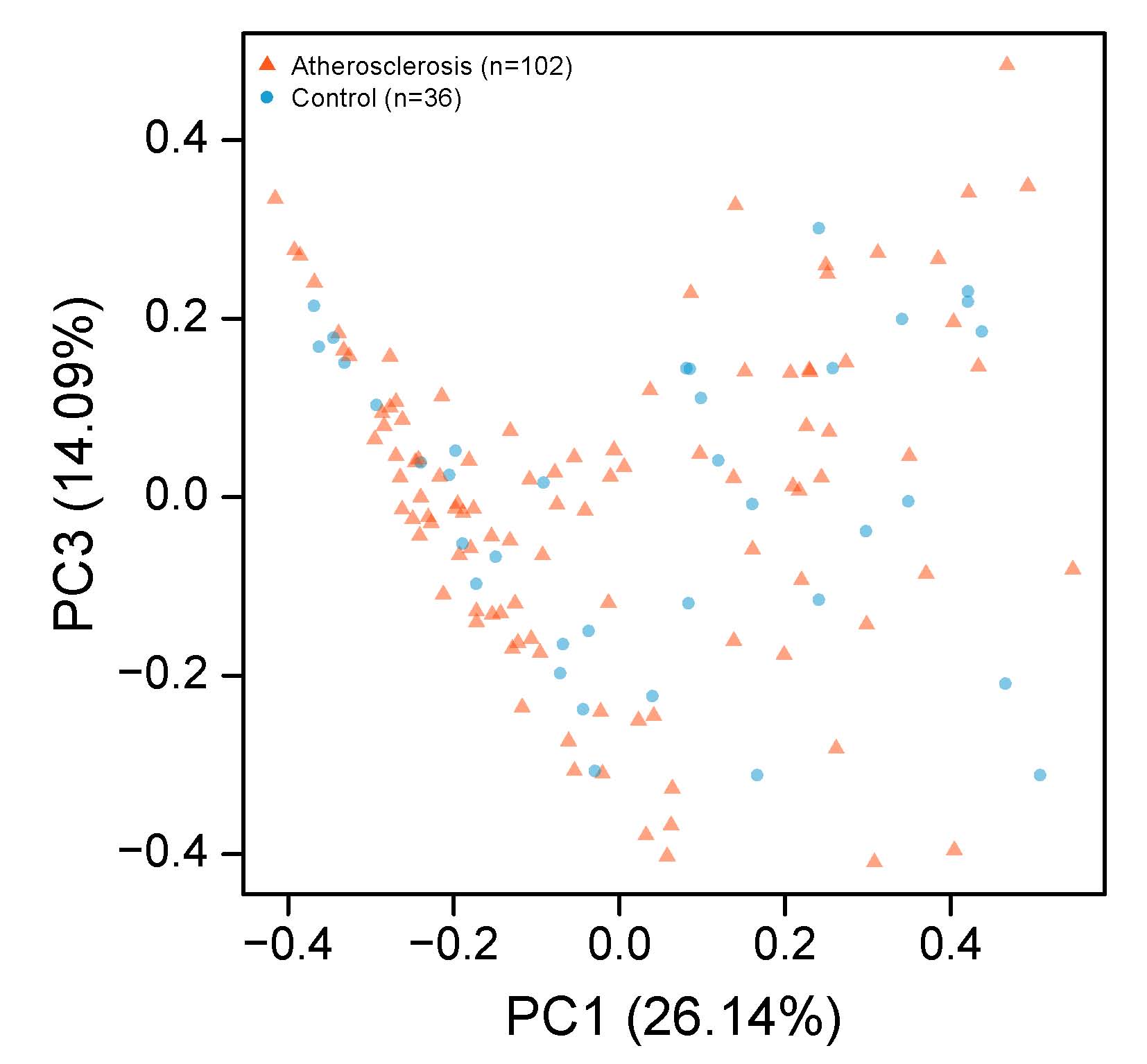

Supplement: Supplementary Figure 7 — PCoA of the genus composition in the gut microbiota. non-CAS, blue circles; CAS, red triangles. [file Image_7.jpeg]

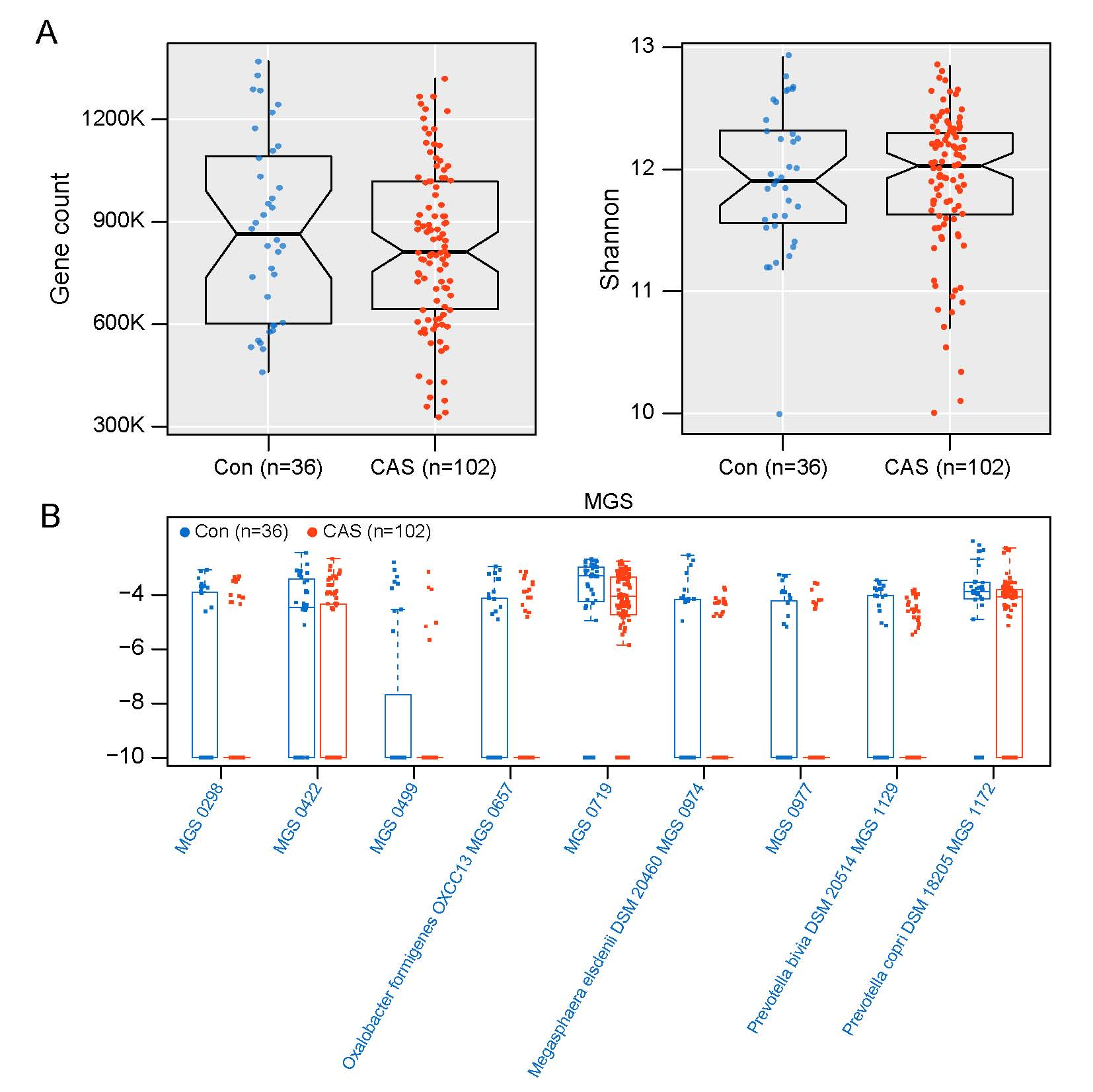

Supplement: Supplementary Figure 8 — Mild alterations of the gut microbiota composition in CAS. (A) Comparison of gene counts (the left panel) and a-diversity (the right panel) of the gut microbiota in non-CAS and CAS individuals by Wilcoxon rank-sum test. (B) MGS differing in abundance between non-CAS and CAS at p<0.05 but q>0.1. [file Image_8.jpeg]

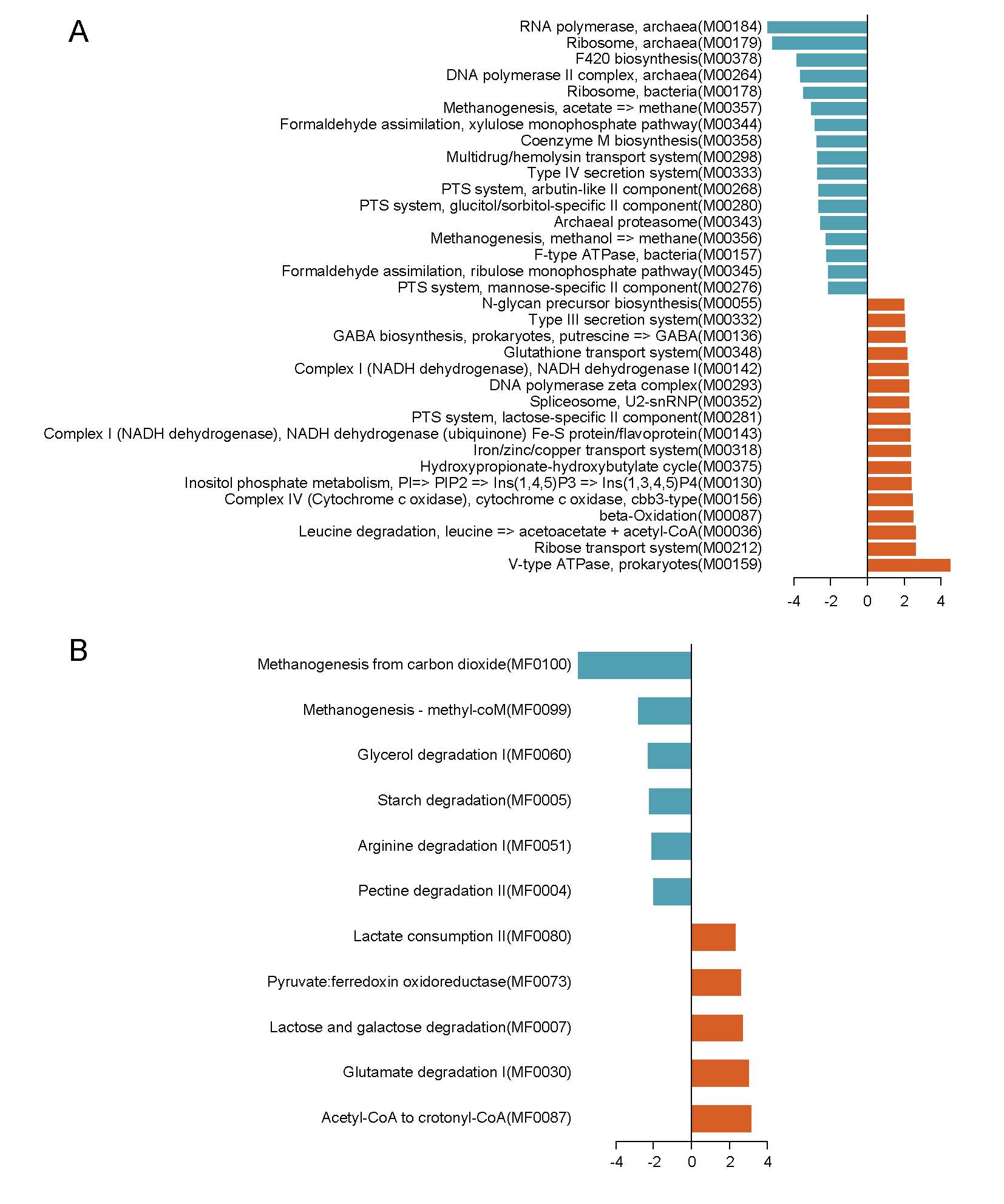

Supplement: Supplementary Figure 9 — Functional alterations of the gut microbiota in CAS. Differentially enriched modules at Z-score>1.6 as analyzed by KEGG (A) and by GMM (B) are shown. Green, enriched in the non-CAS group; red, enriched in the CAS group. Bar length indicates the value of reporter score. [file Image_9.jpeg]
